# Virtual Reality Technology Based on Embedded Image System Simulates Urban Disasters

**DOI:** 10.1155/2022/4741882

**Published:** 2022-03-07

**Authors:** Yuanyuan Wang, Surng Gahb Jahng

**Affiliations:** Graduate School of Advanced Imaging Science, Multimedia & Film in Chung-Ang University, Seoul, 156-756, Republic of Korea

## Abstract

At present, urban disasters are generally valued by various companies and organizations. Although many companies claim to have effectively tested their urban disaster recovery plans, the effectiveness, completeness, and efficiency of their test plans still exist in many aspects. Therefore, it is very important to effectively check the city's disaster recovery plan. Nowadays, both the scientific research community and the industrial community have paid enough attention and research to urban disaster backup. This is a shield of a guaranteed simulation system. The research in this paper belongs to the category of urban disaster virtual reality technology. Urban disaster virtualization is an integral part of virtual reality technology in the current information technology field. Based on the embedded image system, this article divides the virtualization of urban disasters into two meanings: one is to virtualize various disasters into one type of disaster, so as to focus attention on the process and mechanism of disasters on the information system. The meaning is to virtualize different disaster response methods in the standard process, and all links are interrelated and interdependent so that the highest level of disaster response can be implemented. The main goal of disaster virtualization is to integrate different disasters into a comprehensive model. This paper studies urban disasters through embedded image systems and uses virtual reality technology to establish a simulation system to complete the test.

## 1. Introduction

At present, society pays great attention to all aspects of urban disasters, and at the same time, they have carried out preplans [1]. Many companies or organizations have conducted comprehensive tests on urban disaster recovery plans and have a complete set of procedures, but almost half of the tests are unsuccessful [2]. This phenomenon has led to many negative effects such as increased risks, increased costs, and impacts on corporate brands, customer experience, and customer loyalty [3]. Although many companies consider it very important to test urban disaster plans, there are still many IT personnel who have not worked out effective methods to improve the test plan, and one of the ideas for testing urban disaster recovery plans is the urban simulation system and the disaster-to-information system [4]. Since we only focus on the impact of the disaster on the information system, rather than the disaster itself, as long as we know what impact the disaster has on the information simulation system, we can simulate the disaster [5]. In this way, the verification of the disaster recovery plan comes down to the simulation of the disaster [6]. Therefore, the urban disaster simulator designed in this paper relies on virtual reality technology to simulate the impact of fire, earthquake, and network information on the urban system [7]. It is more detailed, more comprehensive, and more effective than direct conversion to verify the disaster backup mechanism [8]. The disaster simulator is divided into three main modules: LAN IP address, disaster evolution module, and disaster on information system impact module. The LAN IP address module uses tools to find IP addresses and select nodes for simulation; the disaster evolution module uses FLEX technology to simulate disaster evolution process, dynamic simulation of the impact of time and space on the outbreak of disasters; the disaster impact module on the information system uses the schtasks statement in the DOS statement, which is intended to connect to the target node remotely, then copy the simulated program, and finally execute it on the target node of the system the disaster simulation program is used to simulate the consequences of a disaster, so as to simulate the impact of the disaster on the information system. The main research work of this paper is to simulate the occurrence, development, and impact of urban disasters on the information system. Then, based on the embedded image system, the evacuation problem is studied [9]. This is an important safety factor in modern society and has attracted attention from all walks of life [10]. Aiming at the evacuation of people in urban disaster emergencies, combined with the uncertain factors in the evacuation process, a multiagent-based evacuation simulation model is established in a multidisaster environment in order to study the laws of people's behavior in disasters [11].

## 2. Related Work

The literature shows the general requirements of disaster simulators and the knowledge and technologies involved in this article, namely, JavaScript, JBoss, JSP, FLEX. The literature proposes a crowd evacuation simulation system in a multihazard environment and conducts a simulation test for the evacuation of the system [12]. The simulation results show that the system better demonstrates the impact of the disaster environment on the evacuation of the people, and the simulation effect on the evacuation is more realistic [13]. It embodies the scientificity and rationality of the crowd evacuation model and establishes a mathematical model based on current disasters [14]. Fire, explosion, and the spread of irritating toxic smoke are the main disasters. This model focuses on the impact of fire and explosion sound on human health and establishes a human perception model and makes it as true as possible based on the actual location of each person. The information is imitated that people can perceive in the environment. The literature shows that FEKO creates a true and false target model in the SAR image, and according to the simulation results, starting from the scattering mechanism, it analyzes the effect of the difference in the geometric structure of the true and false target on the echo scattering [15]. According to the analysis result, a method is proposed. Combining the target feature extraction algorithm of gray and texture, the validity of SAR image authenticity target recognition is determined. The literature shows the definition and characteristics of virtual reality technology and its application advantages in urban disasters, as well as the definition of disaster process exploration mode and disaster processing mode steps [16]. A lot of research on the theoretical basis of virtual city disaster platform research has also been carried out. The literature shows the concept and meaning of unit testing, integration testing, and system testing, and lists some test cases, evaluates the final test results, and compares them with similar solutions and similar products, and finally shows the development and operating environment of the system.

## 3. Embedded Image System and Virtual Reality Technology

### 3.1. Embedded Image System

#### 3.1.1. Embedded System

The development of the embedded system from centralized to a system application platform, open system structure, resource service, intelligent management decision, and green direction is not only a technical upgrade but also meets the needs of practical applications. The system architecture is not only highly modular but also supports reconstruction, modification, and expansion. Chinese universities and scientific research institutes have accumulated some research foundations in embedded systems and reliability calculations, and some scientific research institutions have accumulated rich research experience in embedded systems and microprocessors. Based on the strategy of vigorously developing the advanced manufacturing industry, China has carried out embedded system research in the industrial field and puts forward the requirement of combining intelligence and industrialization. The equipment manufacturing industry is the basic requirement for industrialization. Hardware and software are used in the design of CNC machine tools in the software industry. It is very important in the manufacture of machine parent equipment. CNC system is a typical application of the embedded real-time system in the industrial field.

In foreign countries, the design of embedded systems is also a matter of close concern in academia and industry. Some countries have incorporated the research of embedded systems into the national key strategic research plans and support related research centers.

Now, in the international manufacturing industry, mechanical or electrical components are completely embedded in controlled equipment to create a dedicated computer system designed for specific applications. In the future, embedded systems will develop into cyber-physical systems (CPS) in the industrial field. There will be a large amount of data exchange between sensors, actuators, and computing devices, with the purpose of adapting to different application environments. Data interaction and cooperation are affected by the uncertain factors of the physical world, and it is also a new challenge to the reliability of the system. The design of embedded systems is more inclined to the requirements of low power consumption and higher reliability.

#### 3.1.2. Image Simulation

In the image simulation process, when electromagnetic waves are reflected on the target, a random elevation difference is added to the diameter of the tracking tube to simulate the scattering characteristics of the target surface. On this basis, the combined electromagnetic scattering mode of the target in a complex scene is established as follows:

(1) $$\begin{array}{c} P_{j}^{\prime }=P_{j}+\Delta H_{j}\cdot {\hat{n}}_{i}. \end{array}$$

On the surface of the rail pipe with random elevation, the normal direction of the original reflecting surface is corrected, and *n* ^_*i*^' is the normal direction of the triangle facet after the elevation difference is introduced.

(2) $$\begin{array}{c} {\hat{n}}_{i}^{\prime }=\frac{\left(P_{2}^{\prime }-P_{1}^{\prime }\right)\times \left(P_{3}^{\prime }-P_{1}^{\prime }\right)}{\left(P_{2}\cdot -P_{1}^{\prime }\right)\times \left(P_{3}\cdot -P_{1}^{\prime }\right)}. \end{array}$$

Rayleigh distribution:

(3) $$\begin{array}{c} P_{r}\left(x\right)=\frac{2x}{\alpha }\exp\left(-\frac{x^{2}}{\alpha }\right). \end{array}$$

Lognormal distribution:

(4) $$\begin{array}{c} P_{l}\left(x\right)=\frac{1}{x{\left(\pi \alpha \right)}^{1/2}}\exp\left(-\frac{{\left(\ln\;x-m\right)}^{2}}{\alpha }\right), \end{array}$$

where *m* and *ɑ* represent the mean and variance of lnx, respectively, which can be obtained by maximum likelihood estimation:

(5) $$\begin{array}{c} \left\{\begin{array}{c} \hat{m}=\frac{1}{N}\sum_{i=1}^{N}\ln\left(X_{i}\right)\\ \hat{\alpha }=\frac{2}{N}\sum_{i=1}^{N}{\left[\ln\left(X_{i}\right)-m_{ML}\right]}^{2} \end{array}.\right) \end{array}$$

Weibull distribution:

(6) $$\begin{array}{c} P_{w}\left(x\right)=\frac{\gamma }{\varpi }{\left(\frac{x}{\varpi }\right)}^{\gamma -1}\exp\left[-{\left(\frac{x}{\sigma }\right)}^{\gamma }\right] \end{array}$$

It can be obtained by the maximum likelihood estimation method:

(7) $$\begin{array}{c} \left\{\begin{array}{c} \hat{\gamma }={\left\{\frac{6}{\pi^{2}}\frac{N}{N-1}\left[\frac{1}{n}\sum_{i=1}^{N}{\left(\ln\left(X_{i}\right)\right)}^{2}-{\left(\frac{1}{n}\sum_{i=1}^{N}\ln\left(X_{i}\right)\right)}^{2}\right]\right\}}^{1/2}\\ \hat{\varpi }=\exp\left[\frac{1}{N}\sum_{i=1}^{N}\ln\left(X_{i}\right)+0.5772{\hat{\gamma }}^{-1}\right] \end{array}\right). \end{array}$$

K distribution:

(8) $$\begin{array}{c} P_{k}\left(x\right)=\frac{2}{\alpha \Gamma \left(\mu +1\right)}{\left(\frac{x}{2\alpha }\right)}^{\mu +1}K_{\mu }\left(\frac{x}{\alpha }\right). \end{array}$$

In the case of a large number of sea clutter samples, the second-order and fourth-order methods are the most effective. The results are as follows:

(9) $$\begin{array}{c} \left\{\begin{array}{c} \hat{\mu }=\frac{\left(25/16-M_{5/2}/M_{1/2}M_{2}\right)}{\left(M_{5/2}/M_{1/2}M_{2}-5/4\right)}\\ \hat{\alpha }=\frac{M_{1}}{\sqrt{\pi }\Gamma \left(\mu +1\right)\Gamma \left(\mu +1.5\right)} \end{array}\right), \end{array}$$

where

(10) $$\begin{array}{c} M_{n}=\frac{1}{N}\sum_{i=1}^{N}X_{i}^{n}. \end{array}$$

is the central moment of order *n* of the statistic.

The KS test method is a test method to test whether the data distribution is consistent with the commonly used theoretical distribution. The KS value is the maximum value of the difference between the cumulative empirical data distribution function and the expected cumulative distribution function, denoted by De, used to test the statistics of sample data distribution, where De is expressed as

(11) $$\begin{array}{c} De=\underset{1<i<l}{\max}\left|F\left(x_{i}\right)-F_{l}\left(x_{i}\right)\right|=\underset{1<i<l}{\max}\left|\frac{n}{N}-F_{l}\left(x_{i}\right)\right|. \end{array}$$

KL distance is a measure of information theory. If the assumed distribution is F(*x*_i_) and the actual distribution is *F*_*l*_(*x*_*i*_), the expression of the measure is

(12) $$\begin{array}{c} D\left(F_{l}\left\|\right)F\right)=\int_{i=1}^{N}F_{l}\left(x_{i}\right)\log_{2}\left(\frac{F_{l}\left(x_{i}\right)}{F\left(x_{i}\right)}\right)\text{d}x_{i}. \end{array}$$

For the discrete case, the expression can be simplified to

(13) $$\begin{array}{c} D\left(F_{l}\left\|\right)F\right)=\sum_{i=1}^{N}F_{l}\left(x_{i}\right)\log_{2}\left(\frac{F_{l}\left(x_{i}\right)}{F\left(x_{i}\right)}\right). \end{array}$$

The detection mean square error can be defined as

(14) $$\begin{array}{c} mse=\sum_{i=1}^{N}\frac{{\left[F\left(x_{i}\right)-F_{l}\left(x_{i}\right)\right]}^{2}}{N}. \end{array}$$

Sum the data of all subsegments and average them to obtain the power spectrum of the signal to be estimated, which can be expressed as

(15) $$\begin{array}{c} {\bar{P}}_{per}\left(\omega \right)=\frac{1}{L}\sum_{i=1}^{L}{\hat{P}}_{per}^{i}\left(\omega \right). \end{array}$$

The removal of phase imbalance can be removed by the following formula:

(16) $$\begin{array}{c} I_{rot}=\left[I-Q^{*}\sin\left(\beta \right)\right]sqrt\left(1-\sin\;{\left(\beta \right)}^{2}\right). \end{array}$$

**Figure 1:**
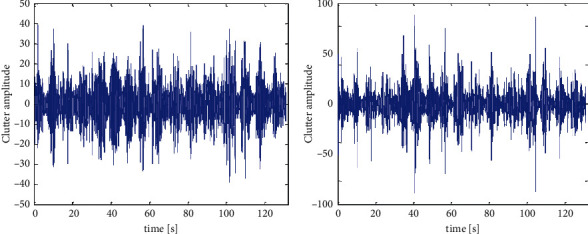
The time series of the amplitude of the seventh range unit with the same polarization.

The actual parameters of the measured scene are sea temperature 7.6°C, wind speed 5.3 m/s, 0° is true north, wind direction 300°, radar appearance direction 125.6°, maximum sea wave height at sea 0.8 m, distance direction 2575–2768 meters, a total of 196 meters, the distance resolution is 30 m, and the target distance tar A is in the seventh unit. The seventh distance unit data is selected as the analysis object.  Figure 1 shows the amplitude-time characteristics of the seventh unit under the same data polarity. The maximum amplitude under VV polarity is 39.38, and the maximum amplitude under HH polarity is 111.5.

**Figure 2:**
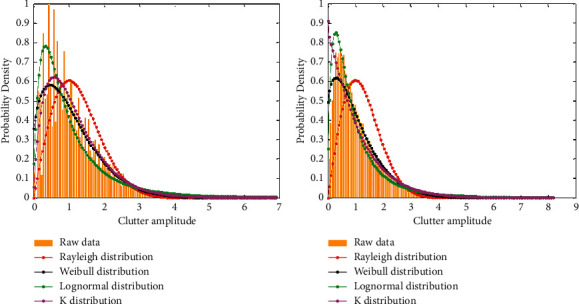
The distribution fitting of the amplitude of the seventh distance unit with the same polarization.

Figure 2 shows the statistical probability density function of the echo amplitude.

Tables 1 and 2 respectively, check the distribution of the distance unit clutter under VV polarity and HH polarity. From the mean value test, under the condition of VV polarization, the clutter amplitude is very suitable for the *K* distribution. In the case of transformation, the amplitude of the clutter fits well with the Weibull distribution.

The clutter distribution test of the seventh distance unit of Document 54 is shown in Table 2:

### 3.2. Virtual Reality Technology

When a fire occurs, the nearby temperature rises and produces flames, smoke particles, and toxic gases. The harmful gases produced by the fire have psychological and physiological effects on the evacuation of people. Since there are many substances produced by fire in practice, the impact on evacuation may be more complicated. Only by simplifying the factors considered in the fire can a corresponding fire model be established. The size of the fire is determined by the power of the fire source, which shows the area of the flame. The diameter *D* and height *L* of the bottom of the fire range can be calculated by

(17) $$\begin{array}{c} D=\sqrt{\frac{4Q}{\pi Q_{e}}},\\ H=-1.02\;D+0.235Q^{2/5}. \end{array}$$

After a fire occurs, smoke is often produced and diffused to the edge, and its diffusion rate is generally determined according to the gas molecule velocity distribution function.

(18) $$\begin{array}{c} V=1.6\sqrt{\frac{RT}{M}}. \end{array}$$

At the initial moment, the concentration and temperature distribution in the air mass are uniform, and the gas concentration is calculated according to the stable continuous point source smoke flow model.

(19) $$\begin{array}{c} C\left(x,y,h,H\right)=\frac{Q}{2\pi u\sigma_{y}\sigma_{z}}\exp\left(-\frac{y}{2\sigma_{y}^{2}}\right)\cdot \left\{\exp\left[\frac{-{\left(h-H\right)}^{2}}{2\sigma_{z}^{2}}\right]+\exp\left[\frac{-{\left(h+H\right)}^{2}}{2\sigma_{z}^{2}}\right]\right\}. \end{array}$$

The steady-state sound pressure level formula is used to calculate the sound pressure level at a certain position.

(20) $$\begin{array}{c} L_{p}=L_{w}+10\mathrm{lg}\left(\frac{Q}{4\pi r^{2}}+\frac{4}{R}\right). \end{array}$$

During the evacuation process, fires often produce smoke and other toxic gases, and other irritating odors may also appear. Pedestrians can perceive the existence of such irritating gases through their sense of smell during travel, which affects people's evacuation routes to a certain extent. Judgment, it also causes certain harm to pedestrians. After a gas leak occurs, people can use the location information and the above equation (18) to calculate the corresponding gas concentration. By obtaining the gas concentration within the range of the person, the human sense of smell can be realized. If the leaked gas is toxic, it will have a significant impact on human physiology. Assuming that the dose of poisonous gas a person bears is S and the probability of causing harm to the person is Ps, the probability formula can be calculated as follows:

(21) $$\begin{array}{c} P_{s}=\frac{1}{\sqrt{2\pi }}\overset{\gamma -5}{\underset{-\infty }{\int }}\exp\left(-\frac{u^{2}}{2}\right)du. \end{array}$$

Among them, the random variable *Y* can appear in the form of a nonlinear equation. When the toxin concentration does not change with time, its toxin value can be calculated as follows:

(22) $$\begin{array}{c} Y=k_{1}+k_{2}In\left(C^{n}t\right). \end{array}$$

From this, a simple calculation formula for the degree of physiological influence *β* can be calculated as follows:

(23) $$\begin{array}{c} \beta =\frac{P_{s}+\theta }{\theta }. \end{array}$$

This article derives a rough calculation following formula for the degree of psychological influence based on empirical data:

(24) $$\begin{array}{c} \alpha =\left(\frac{L_{left}+L_{right}}{2}-120\right)\cdot \sigma . \end{array}$$

## 4. Urban Disaster Simulation System Design and Application Analysis

### 4.1. System Characteristics and Target Analysis

#### 4.1.1. System Analysis

The disaster simulator is mainly used to remotely control the local information system, simulate the impact of the disaster on the information system, and simulate the consequences of the disaster. The key question is how to simulate the impact of the disaster on the information system. In order to accurately simulate the disaster effect, this paper divides the disaster simulator into three modules in the design process: scan to obtain IP address, disaster development module, and simulate the impact of disaster on nodes. First, the network is scanned to obtain the IP address module to achieve the simulation target and delimit the simulation range; that is, the simulator starts; then, the disaster development module can display the changes of the disaster over time and space, truly restore the occurrence and evolution of the disaster, and finally simulate the disaster. The node impact module is the core of the disaster simulator. The simulator creates three states of the information system so that it can truly simulate the impact of the disaster on the information system. The three states are error, malignant change, and crash. Errors indicate the system's failure. The calculation result is incorrect. Malignant change means that the system is running in a random state; crash means that the system is down.

The simulator simulates the impact of disasters on the information system, but only as a simulation. Therefore, security must be considered, and no irreversible impact on the system must be taken into account. In this way, efficiency and security can be achieved at the same time, and a real simulation effect can be achieved.

In addition to its functions, the system must also meet the following characteristics:

Security: Because it is only a disaster simulation, it will not have an irreversible impact on the system. For programs that attack the system.

Clean up after the simulation.

Convenience: Easy to implement and run, the simulator is flexible and convenient, and user operation is simple.

Scalability: Due to technological progress and continuous changes in business conditions, this simulator must have a certain degree of scalability, which can be further expanded and improved.

Efficiency and safety are the basic requirements of this disaster simulator. Convenience and scalability are the highest requirements for the disaster simulator and are the guarantee for improving the quality of the disaster simulator.

After analyzing the functions listed in the requirements, the simulator can be divided into 3 modules.

Automatically, the network is scanned to find the IP address: to obtain the IP address in the local network, you can select the node that simulates the disaster and enters its physical location. Here, you must select the node that needs to be simulated, or you can select all, because sometimes users do not want all the nodes on the local area network. The machines are simulated, so you can choose.

Disaster propagation time and space effects are simulated: the type, intensity, and environmental factors of the disaster are selected to simulate the disaster propagation process. When the disaster spreads to a certain node, the simulator will simulate the impact of the disaster on the system on that node.

Simulation of the impact of disasters on nodes: The consequences of disasters on simulated nodes are realized through remote control. This module is the basic part of the disaster simulator, which can simulate the occurrence, development, and impact of disasters on information systems in detail and truly.

#### 4.1.2. The Goal of the System

The basic use of the disaster simulator is to detect the disaster backup mechanism by simulating the impact of fire, earthquake, and disaster virus on the information system and to simulate the occurrence of disaster and the impact on the information system.

In order to achieve the goal of the disaster simulator to simulate the impact of the disaster on the information system, the simulator must achieve the following effects:

1. Detailed and realistic simulation of the occurrence, development, and impact of disasters on information systems
2. Check the status of the information system after the attack, and check the stability of the system
3. Check the backup mechanism of the information system and provide data for user analysis
4. Check the database backup mechanism, which can be analyzed and improved by the database administrator
5. Check the stability and security of the network
6. Test all users' disaster recovery plans. Only when these overall effects are achieved, the goal of the disaster simulator can be achieved

Many staff members participated in the operation, operation and cooperation of the disaster simulator, the main relevant participants: users, user system administrators, network administrators, database administrators, user system administrators, and disaster backup personnel. The following is a brief introduction of the main personnel.

User: The user needs to use the disaster simulator correctly according to the operation manual, input various effective parameters of the simulation, and clean up the simulated program according to the operation manual.

System maintenance personnel: System maintenance personnel not only analyze its related data but also check the impact on the system.

Network administrator: The network administrator is mainly responsible for two parts: checking and setting up the network, ensuring the normal use of the emulator, and checking the security and stability of the network according to the attack effect of the emulator.

Database administrator: Pay attention to the impact of the simulator on the database. The stability and backup mechanism of the database should be carefully checked.

User system administrator: The user system administrator needs to analyze and check the effect of the simulator, especially to check its security and stability.

Disaster backup personnel: When analyzing the entire operation process, disaster backup personnel monitors and improves their disaster backup mechanism.

The development and design of disaster simulators need to fully consider relevant personnel so that the effects of disaster simulators are more perfect.

### 4.2. Overall System Design

As a traditional tool, the flow chart of the disaster system is mainly used to describe the physical model of the system. It is a graph that uses some graphic symbols to describe the process of the system. It is required to describe various components, such as tables, files, and programs, and display them.

The function of the flow chart of the disaster system is manifested in the following aspects: The flow chart of the disaster system is a tool for business operators, administrators, and system analysts to communicate with each other. Through the flow chart of the entire disaster system, members can communicate well. Another function of the system flow chart is to allow the system analyst to effectively understand the overview of the disaster system business processing, which is also the basis for the disaster system analyst to make further analysis, and it is also the beginning of the system design.

The flowchart system not only understands the entire disaster system but can also analyze the rationality of the business process of the disaster system.

From the analysis of the requirements of disaster software to the completion of the final development, the most important step is the use case diagram. The use case diagram describes how people use the disaster system and shows the relationship between users and what services users need so that users of the system can understand more easily. The purpose of these elements is also convenient for software developers to finally realize these elements. Use case diagrams are generally used by various development activities, many of which are used to describe systems and subsystems. The system can be divided into very specific units, so it has become an excellent tool for software developers.

**Figure 3:**
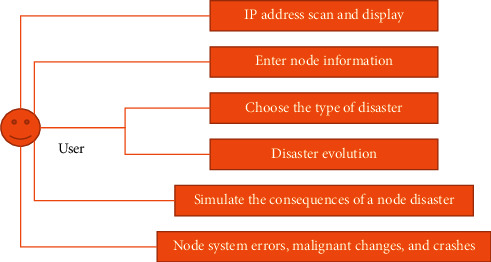
System use case diagram.

As shown in Figure 3, this system mainly has six use cases.

**Figure 4:**
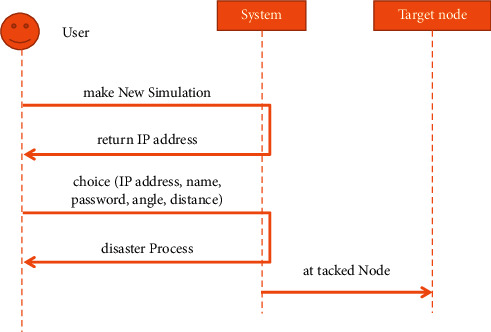
System sequence diagram.

Figure 4 shows the interaction events between the users of the system and the disaster system: first, the system is started, and then, the system returns all the IP addresses of the local area network, and then, the user selects the node, the disaster type, and environmental factors to simulate, and finally, the system simulates the evolution process of the disaster. The system simulates the consequences of implementing a disaster on the node.

The system sequence diagram is a fast and simple product that illustrates the input and output events related to the system in question. The system sequence diagram is the input to the operation contract and object design, which shows the external participants directly interacting with the disaster system, and the participants initiated system events. For some use cases, system sequence diagrams should be drawn for the main success scenarios of each use case, as well as frequently occurring or complex alternative scenarios.

### 4.3. System Function Design

Receiving IP address module: There are four methods for receiving IP addresses, DNS, SNMP, ping command, and Traceroute command, but these contents are not the key issues of the disaster simulator, and we can obtain IP addresses by using tools. There are many such tools and found that these tools are more complicated and confusing, not suitable for disaster simulators. Finally, this article found a simple and practical tool. Local area network IP auto-configurer, its interface, and operation are relatively simple and can be used in the disaster simulator designed in this paper. This module is to start the disaster simulator, find all the IP addresses on the device LAN, and then select the node to be simulated to achieve complete simulation, and the distribution map of the target node can be formulated according to the node selected by the user and input parameters for later dynamic demonstration.

Disaster evolution module: First, you need to use animation technology to simulate the evolution of fires, earthquakes, and computer viruses and make models. The simulator uses FLEX to achieve the simulation effect. Flex uses a non-Flash path to interpret the .mxml files to organize components and create corresponding files. Among them, this article uses FLEX technology to simulate the time and space effects of the disaster spread on the page, that is, dynamically simulate the evolution process of disasters. After the user selects the parameters, the background program formulates the evolution formula according to the parameters, and the page changes according to the evolution simulation to simulate the change of the disaster. For fires, earthquakes, and computer viruses, different evolution models are created to adapt to the different nature of disasters and try to truly simulate the evolution process of disasters in the local area network in reality.

System impact module: The core of the disaster simulator is the system impact module. To start using this module, you need to complete the following steps: (1) To set up a link to each target node, you must first be able to connect to each target node; (2) to create an empty folder in the designated area of the target node, it is best to create this folder on the C drive, because most computers have a C drive; (3) to copy the simulation program to an already created folder; (4) to remotely start the simulation program. The simulation program is mainly composed of bat files, VBScript programs, and enhanced computer code viruses. The process of system error, system malignancy, and system crash must be realized. These steps must be controlled by the background program, adapt to the disaster evolution process, and be able to cover all target nodes. This module is essential for simulating disasters. It must be able to accurately and timely implement the simulation, and it must realistically simulate the consequences of the disaster. Therefore, the simulation program must be designed to be effective and powerful.

**Figure 5:**
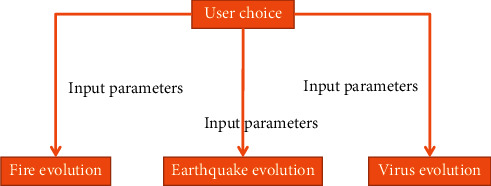
Disaster evolution structure diagram.

The disaster evolution structure is shown in Figure 5.

### 4.4. System Test Analysis

In the development process of the entire disaster system, unit testing is the lowest level of testing. When testing a unit, the unit that needs to be tested is isolated from other parts. Unit testing is repeatable. Test units can be tested repeatedly to make the test results more accurate. Therefore, the maintenance of unit testing cannot exceed the life cycle of all software.

The unit test case style is shown in Table 3.

The main purpose of integration testing is to combine different modules to test to find problems and defects in each interface. If data are not transmitted through the interface, the interface does not have a connection effect; the two modules are integrated together and affect each other, resulting in failure of function realization; after the integration, the module did not achieve the expected function and so on.

The IP address module integration test case is obtained, as shown in Table 4.

System testing is a combination of software, hardware, external equipment, network, interface, and other elements required for system operation, and then, the overall test is performed to verify whether the system functions, performance, and functions meet the requirements of the entire system and to understand which areas are not. The requirements are met, and targeted changes and improvements are made. After testing the system and discovering the problem, it is necessary to be able to locate the problem and know where the problem is, so as to facilitate the improvement of the problem. The system test requirements can cover all the main functions, including not only the test software but also other components such as hardware, external equipment, and networks, interfaces.

Disaster simulator system test cases are shown in Table 5.

## 5. Conclusion

This paper focuses on the simulation of evacuation in an urban disaster environment based on virtual reality technology and establishes an evacuation awareness model. Compared with the research on evacuation based on perception patterns at home and abroad, this paper combines people and the environment and combines the environment and perception evacuation research from the perspective of human perception, and expounds the impact of disasters on evacuation. It also simulates the impact of fires, earthquakes, and information systems on the city. According to the analysis of the impact of urban disasters on the urban information system, the entire framework is designed. The simulator is divided into three functional modules: IP address acquisition, disaster evolution, and the impact of the system. The three modules were designed, developed, and tested in sequence, the three modules were integrated, and the system was tested, and a disaster simulator was deployed. In order to better respond to urban disasters, it is necessary to improve the recommendations and norms of disaster response, and it is urgent to conduct disaster simulation research. This paper has done a lot of research on fire, earthquake, and information system simulation, and this direction will inevitably become a new and valuable research direction.

## Figures and Tables

**Table 1:** Clutter distribution test of the seventh distance unit (VV polarization).

---	Rayleigh distribution	Weibull distribution	Lognormal distribution	K Distribution
K-S inspection	0.9247	0.9247	1.7646	0.7875
K-L inspection	13.0163	17.1023	17.1412	8.9692
MES inspection	0.0171	0.0083	0.0097	0.0073
Test mean	3.4896	4.5089	4.7289	2.4408

**Table 2:** Clutter distribution test of the seventh distance unit (HH polarization).

---	Rayleigh distribution	Weibull distribution	Lognormal distribution	K Distribution
K-S inspection	0.5978	0.5889	0.9922	1.5817
K-L inspection	14.7828	0.8135	0.8952	2.0242
MES inspection	0.0162	0.0019	0.0015	0.0067
Test mean	3.8493	0.3534	0.4722	0.9032

**Table 3:** Test case style.

Test case number	Test-01
Test item	Disaster simulator
Test title	Test of the disaster simulator
Importance level	High
Parameter input	Test
Execution steps	—
Expected result	Achieve the desired goal

**Table 4:** Test case IPA-001.

Test case number	diSimulator-IPA-001
Test item	Disaster simulator
Test title	Perform integration test on the module for obtaining the IP address
Importance level	High
Prefabricated conditions	—
Parameter input	Start IP address, end IP address
Execution steps	Automated tool execution
Expected result	Obtain all IP addresses in the selected segment of the LAN

**Table 5:** Test case T-001.

Test case number	diSimulator-T-001
Test item	Disaster simulator
Test title	System test of disaster simulator
Importance level	High
Prefabricated conditions	Select multiple simulation nodes
Parameter input	Choose fire, earthquake, computer virus separately, choose different intensity, choose different environmental factors
Execution steps	—
Expected result	All selected nodes eventually crash

## Data Availability

The data used to support the findings of this study are currently under embargo while the research findings are commercialized. Requests for data, after the publication of this article, will be considered by the corresponding author.
